# Impact of a dissemination strategy on family day care educators’ intentions to adopt outdoor free play guidelines introduced in response to COVID-19: a randomized controlled trial

**DOI:** 10.1093/her/cyad014

**Published:** 2023-03-21

**Authors:** M Lum, S L Yoong, L Wolfenden, H Turon, K Reilly, A Grady

**Affiliations:** Hunter New England Population Health, Hunter New England Local Health District, Booth Building, Longworth Ave, Wallsend, NSW 2287, Australia; School of Medicine and Public Health, University of Newcastle, University Dr, Callaghan, NSW 2308, Australia; Hunter Medical Research Institute, University of Newcastle, Lot 1 Kookaburra Cct, New Lambton Heights, NSW 2305, Australia; Priority Research Centre for Health Behaviour, University of Newcastle, University Dr, Callaghan, NSW 2308, Australia; Hunter New England Population Health, Hunter New England Local Health District, Booth Building, Longworth Ave, Wallsend, NSW 2287, Australia; School of Medicine and Public Health, University of Newcastle, University Dr, Callaghan, NSW 2308, Australia; Hunter Medical Research Institute, University of Newcastle, Lot 1 Kookaburra Cct, New Lambton Heights, NSW 2305, Australia; Priority Research Centre for Health Behaviour, University of Newcastle, University Dr, Callaghan, NSW 2308, Australia; Global Centre for Preventive Nutrition and Health, Institute for Health Transformation, School of Health and Social Development, Deakin University, 221 Burwood Highway, Burwood, VIC 3125, Australia; Hunter New England Population Health, Hunter New England Local Health District, Booth Building, Longworth Ave, Wallsend, NSW 2287, Australia; School of Medicine and Public Health, University of Newcastle, University Dr, Callaghan, NSW 2308, Australia; Hunter Medical Research Institute, University of Newcastle, Lot 1 Kookaburra Cct, New Lambton Heights, NSW 2305, Australia; Priority Research Centre for Health Behaviour, University of Newcastle, University Dr, Callaghan, NSW 2308, Australia; School of Medicine and Public Health, University of Newcastle, University Dr, Callaghan, NSW 2308, Australia; Hunter Medical Research Institute, University of Newcastle, Lot 1 Kookaburra Cct, New Lambton Heights, NSW 2305, Australia; Priority Research Centre for Health Behaviour, University of Newcastle, University Dr, Callaghan, NSW 2308, Australia; School of Medicine and Public Health, University of Newcastle, University Dr, Callaghan, NSW 2308, Australia; Hunter Medical Research Institute, University of Newcastle, Lot 1 Kookaburra Cct, New Lambton Heights, NSW 2305, Australia; Priority Research Centre for Health Behaviour, University of Newcastle, University Dr, Callaghan, NSW 2308, Australia; Hunter New England Population Health, Hunter New England Local Health District, Booth Building, Longworth Ave, Wallsend, NSW 2287, Australia; School of Medicine and Public Health, University of Newcastle, University Dr, Callaghan, NSW 2308, Australia; Hunter Medical Research Institute, University of Newcastle, Lot 1 Kookaburra Cct, New Lambton Heights, NSW 2305, Australia; Priority Research Centre for Health Behaviour, University of Newcastle, University Dr, Callaghan, NSW 2308, Australia

## Abstract

In 2021, guidelines for early childhood education and care were released recommending children are provided access to outdoor areas during all free play sessions to reduce the risk of coronavirus disease of 2019 transmission, aligning with the existing recommendations to increase children’s physical activity. There is a need to understand how to disseminate guidelines in this setting as dissemination is a prerequisite of adoption and implementation. This randomized controlled trial explored the impact of a video-based strategy to disseminate guidelines on family day care educators’ intentions to adopt outdoor free play guidelines. Educators (*N* = 255) were randomized to receive a video (intervention) or text-based (usual care) resource via email describing recommendations. Educators were invited to participate in a post-intervention survey at 5-week follow-up assessing intentions to adopt guidelines. The secondary outcomes included knowledge, beliefs about capabilities, beliefs about consequences, social/professional role and identity, goals, implementation of guidelines, acceptability of resource and intervention reach. There was no statistically significant difference between groups in intentions to adopt guidelines [ß = 0.01 (95% confidence interval −0.50 to 0.52), *P* = 0.97], nor for any secondary outcomes. Further investigation is needed to identify effective dissemination strategies in the family day care setting to increase the adoption of public health guidelines.

## Introduction

Adequate physical activity in young children is associated with reduced risk of overweight and obesity, as well as improved motor skills, fitness and skeletal health [1, 2]. Global guidelines recommend that children <5 years accumulate 180 minutes of physical activity every day [3], however systematic review evidence indicates that many children internationally do not meet this benchmark [4–6]. Given early childhood education and care (ECEC) services have existing infrastructure to promote physical activity and reach up to 87% of children aged 3–5 years for prolonged periods of the day [7], the World Health Organization recommends ECEC as a key setting to improve children’s physical activity [8].

ECEC services include centre-based services, such as preschools and long day care, and home-based services, known as family child care homes, childminding or family day care (as referred to hereafter). In comparison to centre-based services which have multiple staff members employed in various roles (e.g. nominated supervisor, educator, director and service cook), family day care is a type of regulated ECEC service in which an individual educator provides care to small numbers of children in the educator’s own home [9]. In Australia, family day care services are monitored and supported by an overarching organization (i.e. service provider/scheme); however, they are predominantly responsible for their services’ operation [10]. Family day care is accessed by >90 000 children for an average of 25 hours per week in Australia [11].

Systematic review evidence of observational studies indicates that outdoor play in ECEC services is associated with increased physical activity levels and reduced sedentary behaviour in children [12, 13]. These findings are also supported by evidence from recent randomized controlled trials (RCTs) [14, 15]. As such, many authorities internationally recommend ECEC services, including family day care, provide children with outdoor play every day [16]. Despite the potential benefits of offering outdoor play to children, a cross-sectional study published in 2022 suggests that just 24% of family day care services provide at least 60 minutes of outdoor play time for children per day [17]. Recently, government authorities released public health guidelines which recommend children spend more time outside to reduce the risk of transmission of coronavirus disease of 2019 (COVID-19) in ECEC [18, 19], aligning with the best practice physical activity recommendations [16]. Therefore, this provides an opportunity to understand how to best support dissemination of these guidelines to family day care educators.

Several implementation theories suggest that the dissemination of public health guidelines to end users is an important prerequisite to achieving adoption and implementation [20–22]. Effective dissemination requires identifying an appropriate audience and delivering a tailored message and medium that best targets the barriers to adoption in this particular audience [23]. A RCT in centre-based ECEC services found evidence that targeted dissemination via enhanced resources improves ECEC service adoption of public health guidelines [24]. Given the structural differences in centre-based and family day care services (e.g. educators are individually responsible for their services’ daily programming), barriers to guideline adoption and implementation may differ. In particular, barriers pertinent to an individual’s knowledge and beliefs (e.g. capability of implementing, consequences to implementing, alignment of professional role and goals) may need to be addressed specifically within dissemination strategies in this setting [25]. To date, no known trials have assessed the impact of different dissemination strategies on increasing intentions to adopt guidelines in the family day care setting.

## Aims

The primary aim of this RCT was to explore the impact of guideline dissemination via a video resource (intervention) in comparison to a text-based resource (usual care control) sent by email on family day care educators’ intentions to adopt outdoor free play guidelines. It was hypothesized that family day care educators’ intentions to adopt guidelines would be higher in the intervention group, compared to control, as a result of viewing the video resource.

The secondary outcomes included selected Theoretical Domains Framework (TDF) constructs [26] of knowledge, beliefs about capabilities, beliefs about consequences, social/professional role and identity and goals; implementation of guidelines and acceptability of resource format. It was hypothesized that TDF constructs [26], implementation of guidelines and acceptability of the resource format would be higher in the intervention group, compared to control.

As dissemination via video resources has not been explored in the family day care literature, we also assessed the reach of the video (intervention) resource.

## Materials and Methods

Ethical approval was obtained from Hunter New England [2019/ETH12353 (84943)] and the University of Newcastle (H-2008-0343) Human Research Ethics Committees. The trial methods, design and outcomes were prospectively deposited with the Open Science Framework (10.17605/OSF.IO/J6YRD). Participants were provided an information statement for the study and advised that all information collected would be treated confidentially and only accessible by authorized members of the research team. Participants provided informed consent to participate.

## Context

In July 2021, guidelines from the New South Wales (NSW) Department of Education were released to reduce the spread of COVID-19 in ECEC services [19]. These included the recommendation for ECEC services, including family day care services, to consider allowing children to access outdoor areas during all free play sessions, wherever possible [19].

## Design and participants

A RCT design with post-intervention data collection only was employed. Registered family day care educators who care for children aged 0–12 years and operated a service within the Hunter region of NSW were eligible to participate in this study. A previous study [27] indicates that there are >500 family day care educators in the Hunter region, the majority of whom identify as women (100%) and work in low socioeconomic status (SES) (70%) and urban (53%) areas [27]. Non-English-speaking educators and those catering solely to children with additional needs were ineligible. Educators’ contact details were obtained from service providers/schemes (overarching manager organization) collected for a previous study [27] and from a professional development event held by ‘Good for Kids’ in 2019. ‘Good for Kids’ is an initiative in the jurisdiction which has supported ECEC services to promote healthy eating and physical activity in children since 2006 [28] and is a known and trusted source of advice for family day care services.

## Randomization and blinding

Randomization of family day care educators to intervention or control groups occurred at a 1:1 ratio using a random number generator in Microsoft Excel. This was an open trial, and educators were aware that they had received the intervention. Data collectors (those collecting survey data) could not be blinded to allocation due to the intervention group receiving additional questions regarding the video.

## Intervention

Intervention development was informed by the Model for Dissemination of Research which describes four key components required for effective dissemination: source, audience, channel and message [20]. We predefined the source (i.e. ‘Good for Kids’, a reputable organization trusted by the target audience) and audience (i.e. family day care educators, as delivery system actors in a key setting for implementation), which remained constant across both groups. Therefore, this trial sought to manipulate the dissemination channel and message. In regard to channel, qualitative research indicates that ECEC staff prefer to receive information through video messages (as they are engaging) delivered via electronic media [29]. Furthermore, Brownson and colleagues note that dissemination ‘is enhanced when messages are framed in ways that evoke emotion and interest and demonstrate usefulness’ and identify YouTube as a useful tool for non-research audiences [20]. As such, a video resource available on YouTube was selected as the intervention channel. To manipulate the message, we included content which addressed selected TDF constructs [26] (i.e. knowledge, beliefs about capabilities and consequences, social/professional role and identity and goals) as these are identified barriers to guideline adoption and implementation and amenable to change via strategies embedded within a resource [25, 26]. Examples of content included simple, achievable steps and examples of how to implement guidelines (targets knowledge, beliefs about capabilities and goals) and the multiple benefits of implementing guidelines as a family day care educator (targets beliefs about consequences, social/professional role and identity).

The intervention group received an email from ‘Good for Kids’ in March 2022 which included a web link to the video resource on YouTube (accessible by email invitees only to prevent contamination) and a web link to the guidelines on the NSW Department of Education website [19]. The video resource was designed by implementation researchers and health promotion staff and created using Powtoon software [30]. As per the best practice suggestions [31], the video contained good audio and visual quality, was short (2 minutes) and presented a clear message using simple language, with only essential information included. The video was tailored to the target audience by including first person narrative, spoken from the perspective of a family day care educator; dynamic, colourful imagery of children to represent the family day care setting and evoke emotion and practical, setting-specific tips for the target behaviour (i.e. providing more outdoor play) which were visually demonstrated. The 2-minute video has been made publicly available following all data collection and analysis for this study (goodforkids.nsw.gov.au/early-childhood-services/resources/physical-activity).

## Control group

The control group received usual care which consisted of an email from ‘Good for Kids’ in March 2022 containing a generic text-based description of the guidelines only and a web link to the guidelines on the NSW Department of Education website [19].

## Data collection procedure and measures

All potential participants were sent an email from ‘Good for Kids’ ∼5 weeks after the initial email (April 2022) inviting them to participate in a 10-minute online survey. Non-responders were sent a reminder email and followed up with a phone call by a trained interviewer after 2 weeks to invite survey completion via telephone. A minimum of 2 and a maximum of 10 phone contacts were attempted for non-responding educators. Data collection was finalized in August 2022.

### Service and educator characteristics

Items to assess service and educator characteristics included age groups of children cared for, number of hours and days of service operation, number of children attending each day, number of children enrolled from culturally and linguistically diverse backgrounds, highest relevant qualification and service postcode to determine service SES (high or low) and locality (urban or rural).

### Primary outcome

#### Intentions to adopt guidelines

Four items, validated and previously used by the research team in centre-based services [32], were adapted to assess educators’ intentions to adopt guideline recommendations (see Supplementary file). Items were measured on a 7-point Likert scale and scored as: 1 =‘strongly disagree’; 2 =‘disagree’; 3 =‘slightly disagree’; 4 =‘neither agree nor disagree’; 5 =‘slightly agree’; 6 =‘agree’ and 7 =‘strongly agree’. For example, ‘I intend to offer more outdoor play time according to the guidelines at every opportunity.’

### Secondary outcomes

#### *Selected TDF constructs* [26]

A tool to assess TDF constructs [26] has previously been developed and validated by the research team for use in the ECEC setting [32]. Items from this tool were adapted and used to assess knowledge (2 items), beliefs about capabilities (3 items), beliefs about consequences (3 items), social/professional role and identity (3 items) and goals (2 items). Items were scored on a seven-point Likert scale as aforementioned (see Supplementary file).

#### Implementation of guidelines

Four items previously used by the research team in the ECEC setting [27, 33] were used to assess implementation of the guidelines (i.e. allowing children to access outdoor areas during all free play sessions) following the intervention. Items included ‘Does your service provide unstructured child-initiated free play?’ (yes/no response) and ‘Based on the last week, how much time in total is provided for unstructured child-initiated free play per day?’; ‘Of the total time provided for child-initiated free play, how much of that time was for indoor free play per day?’ and ‘Of the total time provided for child-initiated free play, how much of that time was for outdoor free play per day?’ (responses in minutes). Services were classified as ‘implementing’ the guidelines if all free play time allowed children to access the outdoor areas, consistent with recommendations within the resource.

#### Acceptability of resource

Four items based on the validated Acceptability of Intervention Measure [34] were used to assess the acceptability of the resource (i.e. video or text-based) on a 5-point Likert scale, where 1 =‘completely disagree’; 2 =‘disagree’; 3 =‘neither agree nor disagree’; 4 =‘agree’ and 5 =‘completely agree’. For example, ‘I like communication via [video/text-based] messages’.

#### Reach of the intervention resource

Educators in the intervention group were asked whether they had viewed the video resource related to the guidelines (one item). Analytical data were obtained from YouTube [35] to measure the number of views, average watch time duration and number of complete views of the video.

## Sample size calculation

Contact details of 255 potentially eligible family day care educators were available. Assuming a consent rate of ∼30%, a sample of 72 (36 per group) would allow for a detectable difference of 0.4 mean score difference between groups for the primary outcome (intentions to adopt guidelines) and an SD of 0.6 (based on a previous study) [24] with a power of 80% at alpha set at *P* = 0.05.

## Statistical analysis

Statistical analyses were undertaken using Stata IC/14.2. Services were classified as ‘high’ SES if they resided in postcodes ranked in the top 50% and ‘low’ SES if they resided in postcodes ranked in the bottom 50%, according to the Socioeconomic Indices for Areas for NSW [36] scores. Services were classified as ‘urban’ (inner regional or major cities) or ‘rural’ (in outer regional, remote or very remote) according to the Australian Standard Geographical Classification [37]. Consenter and non-consenter characteristics were compared using Pearson’s chi-squared test. Descriptive statistics were generated to describe service and educator characteristics. Service and educator characteristics were compared between groups using Pearson’s chi-squared test. For intentions to adopt and each TDF construct [26] assessed, a mean score was calculated by combining items related to each construct (range: 1–7). Similarly, a mean score was calculated for acceptability using the four items to assess this outcome (range: 1–5). Intention-to-treat linear regression analyses were employed to assess the difference in outcomes between groups, controlling for SES and locality for all outcomes (as per trial protocol). As the study obtained follow-up data only (i.e. no baseline data were collected), if a responding participant did not complete all items relevant for an outcome (e.g. if they only answered three of the four items used to assess intentions to adopt), data were excluded in the analysis of that outcome only. Responding participants with some missing data were still included in analyses of other outcomes where full data were available. All statistical tests were two-tailed, with significance value set at 0.05.

## Results

**Fig. 1.**
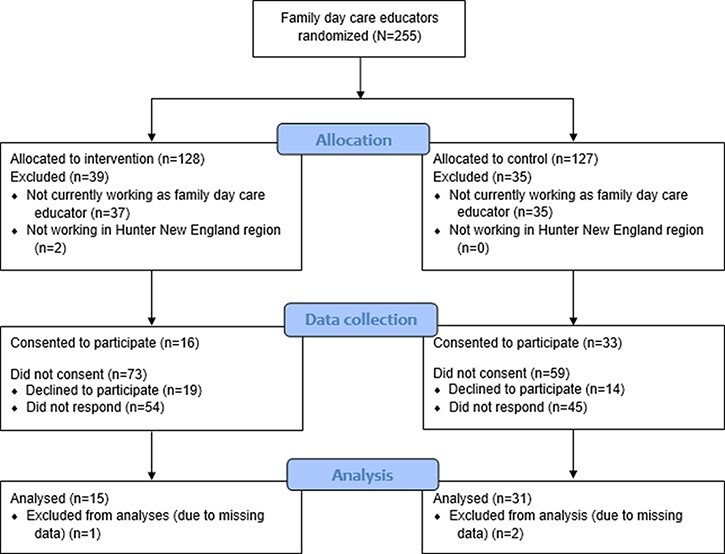
Figure CONSORT flow diagram.

Of the 255 family day care educators, 74 (29.0%) were ineligible (Fig. 1). There was a statistically significant difference in consent rates among educators allocated to the intervention group (18.0%), compared to control (35.9%, *P* = 0.007). There were no statistically significant differences found between consenters and non-consenters for SES (χ^2^ = 0.11, *P* = 0.74) or locality (χ^2^ = 1.5, *P* = 0.22).

### Characteristics of consenters

Service and educator characteristics are described in Table I. There were no statistically significant differences in characteristics between the intervention group and control. Services cared for four children per day on average, with almost all services (98.0%) caring for children aged 3–6 years. The majority of services were located in low SES (79.6%) and urban (81.6%) areas.

**Table I.** Characteristics of intervention and control consenters

Characteristics	Total (*N* = 49)	Intervention (*n* = 16)	Control (*n* = 33)
Number of children attending per day (mean, SD)	4.3 (0.9)	4.4 (1.0)	4.3 (0.8)
Service cares for children from culturally and linguistically diverse backgrounds (*n*, %)	22 (44.9)	5 (31.3)	17 (51.2)
Service cares for children aged 3–6 years (*n*, %)	48 (98.0)	16 (100)	32 (97.0)
Service opens 5 days per week (*n*, %)	21 (42.9)	5 (31.3)	16 (48.5)
Service open ≥8 hours per day (*n*, %)	40 (81.6)	15 (93.8)	25 (75.8)
Service SES, high (*n*, %)	10 (20.4)	3 (18.8)	7 (21.2)
Service locality, urban (*n*, %)	40 (81.6)	14 (87.5)	26 (78.8)
Educator holds Cert. IV qualifications or higher (*n*, %)^a^	17 (35.4)	12 (75.0)	5 (15.6)

a *N* = 48 due to missing data.

### Primary and secondary outcomes

There were no statistically significant differences between groups on mean scores of educators’ intentions to adopt guidelines {ß = 0.01 [95% confidence interval (CI) −0.50 to 0.52], *P* = 0.97} (Table II). There were no statistically significant differences between groups on mean score outcomes for any of the TDF constructs [26] assessed (Table II).

**Table II.** *Family day care educator’s mean scores for intentions to adopt guidelines, TDF constructs* [26] *and percentage of time family day care educators implemented guidelines (N= 46)*

	Intervention		Control			
Outcome	*N*	M (SD)	*N*	M (SD)	Effect size (95% CI)	*P*
Intentions to adopt	15	6.03 (1.01)	30	5.99 (0.73)	0.01 (−0.50, 0.52)	0.97
Knowledge	14	6.36 (0.95)	31	6.37 (0.53)	−0.14 (−0.55, 0.28)	0.51
Beliefs about capabilities	15	5.93 (0.82)	30	5.78 (0.85)	0.16 (−0.35, 0.70)	0.51
Beliefs about consequences	15	6.02 (0.66)	31	5.94 (0.98)	0.87 (−0.46, 0.64)	0.75
Social/professional role and identity	14	6.21 (0.71)	30	6.33 (0.56)	−0.13 (−0.54, 0.28)	0.51
Goals	15	5.80 (0.90)	28	5.50 (0.97)	0.26 (−0.35, 0.87)	0.40
Implementation of guidelines	14	84.33 (31.34)	31	75.24 (23.18)	8.30 (−8.84, 25.45)	0.33

#### Implementation of guidelines

There was no statistically significant difference found between groups for the percentage of time educators allowed children to access the outdoor play areas (*P* = 0.33) (Table II). There was no statistically significant difference in the proportion of educators ‘implementing’ the guidelines (i.e. allowing access to outdoor areas during all free play sessions) between the intervention group (56.3%) and control (36.4%, *P* = 0.44).

#### Acceptability of the resource

There was no statistically significant difference between the intervention group (M 4.1; SD 0.5) and the control (M 4.4; SD 0.4) in the acceptability scores of the resource (*P* = 0.08).

#### Reach of the intervention resource

Of the 16 consenting educators in the intervention group, 11 (68.8%) reported viewing the video. Analytical data from YouTube [35] indicated that the intervention resource had 19 unique views. The average view duration was 1 minute and 42 seconds or 85% of the video length. The proportion of viewers who watched the video in its entirety was 62.7%.

## Discussion

This study aimed to assess the impact of a video resource, compared to a text-based resource, on family day care educators’ intentions to adopt outdoor free play guidelines. Despite use of an engaging medium (as described by ECEC staff) [29], keeping video duration in line with recommendations for this format [31] and efforts to tailor the video resource to the target audience and address barriers [25], we found no statistically significant difference between the intervention group and control for any of the primary or secondary outcomes. We found that intentions to adopt were high among both groups, suggesting there may be ceiling effects on the measure used to assess the primary outcome. The smaller than intended sample size is also likely to make it challenging to describe differences between groups.

Previous findings from RCTs which examine dissemination strategies on educators’ intentions to adopt guidelines in the ECEC setting are mixed, with one study demonstrating significant improvements [24] and another reporting no impact [38]. Evidence in centre-based ECEC has indicated that offering follow-up support, training, feedback and incentives in addition to providing video and text-based resources may improve service adoption of the best practice guidelines [39]. In the current trial, control services received usual care, which involved receiving guidelines in generic text-based form. As the intervention only manipulated the channel and message of dissemination, it is possible that the strategies embedded within the video resource content did not sufficiently target the reported barriers to produce notable differences between groups in intentions to adopt guidelines. Furthermore, we were unable to recruit the desired sample of 72 educators which reduced our ability to detect a mean difference of 0.4 mean score. However, the magnitude of effect in the current study suggests that an increased number of participants are unlikely to change the direction of effect found in this study. The recruitment of family day care educators into trials has previously been noted as a challenge [40]. Future trials in this setting should aim to implement strategies to increase study consent rates. This may include engaging with service providers/schemes and having them introduce and endorse the study via newsletters or internal emails prior to recruitment or addressing educators’ concerns about participation during recruitment [40].

We found no statistically significant differences between groups on TDF constructs [26] nor implementation of guidelines (although this favoured the intervention arm), consistent with previous research undertaken in centre-based ECEC settings [41, 42]. Across both groups and all TDF constructs [26], scores were skewed toward the high end of the scale (mean score: 6.0; median score: 6). As the guidelines were publicly accessible prior to the study being conducted, baseline knowledge and beliefs may have reached a peak prior to the dissemination of the video resource and a ceiling effect incurred. While the tool used to assess TDF constructs was validated [32], efforts to improve sensitivity may increase the tools’ ability to detect differences between groups. This may be achieved by removing unnecessary anchors such as ‘slightly agree’, as these may create noise and reduce item comprehensibility, or removing items that are redundant or poorly functioning [43].

Despite high intentions to adopt and awareness of the guidelines, few services reported implementing the guidelines fully, findings consistent with other services in ECEC [44]. Our findings suggest that dissemination strategies may need to be coupled with additional strategies where feasible to increase impact of guidelines [42]. While non-significant, given acceptability of the resource favoured the control, future dissemination efforts may also consider embedding strategies within alternate text-based resources (e.g. interactive newsletters), which has previously been demonstrated to increase intentions to adopt guidelines in centre-based services [24]. There is also RCT and cross-sectional evidence which demonstrates a positive association between having a physical activity policy with outdoor play components and the provision of outdoor play in ECEC services [45, 46]. Future research should consider including strategies to support the development of a service physical activity policy, which aligns with national recommendations to increase the provision of outdoor free play.

This is the first known trial to examine the impact of dissemination of guidelines via a video resource in the family day care setting. While this trial did not produce the expected changes in intentions to adopt guidelines or TDF constructs, there may be benefits in including video as a channel to deliver health information to family day care educators. Our finding that 69% of participants viewed the video resource is comparable to findings reported in a RCT with family day care services in the United States [47]. This suggests that videos may have the capacity to reach the majority of family day care educators and be a useful way to communicate information, which is particularly important in this difficult-to-reach setting [40]. As such, the impact of video delivery of resources to improve child physical activity in the family day care setting should be further explored.

### Strengths and limitations

Strength of this study was the use of a RCT design and application of theory to develop the intervention resource. However, this study had several limitations. There was a significant difference in the number of intervention and control educators consenting to participate. As participants in the intervention group were asked whether they had viewed the video, this may have resulted in selective attrition from the study due to not viewing the video. The target sample size of 72 educators was not reached, likely given the high proportion (29%) of ineligible educators, primarily due to no longer working as a family day care educator. This is unsurprising given the context in which the study was undertaken and the decrease in number of operating family day care services during the pandemic as a result of multiple lockdowns [11]. Furthermore, post-intervention data only were collected; therefore, potential differences in baseline outcomes were not able to be assessed. Finally, data were collected ∼5 months following the delivery of the intervention due to challenges with reaching family day care educators during this time. As the number of participants reporting viewing video was lower than the unique views obtained from analytics, there may have been some recall bias in response given the time lapsed following intervention delivery.

## Conclusion

This study found that a dissemination strategy (resource changing the message and channel components) did not produce differences in educators’ intentions to adopt outdoor free play guidelines compared to control, nor any of the other secondary outcomes assessed (knowledge, beliefs about capabilities, beliefs about consequences, social/professional role and identity and goals; implementation of guidelines and acceptability of resource format). Further research that is adequately powered, using rigorous study designs, is required to understand the impact of different disseminations approaches on increasing the adoption of public health guidelines in the family day care setting.

## Supplementary Material

cyad014_Supp
