# Supplementary material for: Impact of a dissemination strategy on family day care educators’ intentions to adopt outdoor free play guidelines introduced in response to COVID-19: a randomized controlled trial
Source: Health Educ Res. 2023 Mar 21;38(5):458–68. doi: 10.1093/her/cyad014 (PMC10516333; doi:10.1093/her/cyad014)
Supplement: cyad014_Supp [file cyad014_supp.zip › Supplementary file.docx]

**Supplementary file**

Items were adapted from a validated Theoretical Domains Framework Tool^32^ to assess intentions to adopt, knowledge, beliefs about consequences, beliefs about capabilities, social/professional role and identity and goals.

All items were measured on a scale of: 1=‘strongly disagree’; 2=‘disagree’; 3=‘slightly disagree’; 4=‘neither agree nor disagree’; 5=‘slightly agree’; 6=‘agree’; and 7=‘strongly agree’.

Supplementary Table 1. Items adapted from a validated Theoretical Domains Framework Tool^32^ to assess selected Theoretical Domains Framework constructs

| **Intentions to adopt** | 1. I intend to offer more outdoor play time according to the Guidelines in the next six months. |
| --- | --- |
|  | 1. I intend to offer more outdoor play time according to the Guidelines at every opportunity. |
|  | 1. I have a strong intention to offer more outdoor play time according to the Guidelines, at every opportunity. |
|  | 1. I will definitely offer more outdoor play time according to the Guidelines, at every opportunity. |
| **Knowledge** | 1. I am aware of the objectives of the Guidelines. |
|  | 1. I know what my responsibilities are, with regard to offering more time outdoors according to the Guidelines. |
| **Beliefs about consequences** | 1. I believe offering more time outdoors according to the Guidelines, will benefit public health. |
|  | 1. I believe offering more time outdoors according to the Guidelines, will lead to benefits for the children. |
|  | 1. In my view, offering more time outdoors according to the Guidelines, is practical. |
| **Beliefs about capabilities** | 1. I am confident that I can offer more time outdoors according to the Guidelines. |
|  | 1. I am capable of offering more time outdoors according to the Guidelines, even when little time is available. |
|  | 1. For me, offering more time outdoors according to the Guidelines, is easy. |
| **Social/professional role and identity** | 1. Offering more time outdoors according to the Guidelines is part of my role. |
|  | 1. It is my responsibility to offer more time outdoors according to the Guidelines. |
|  | 1. Offering more time outdoors according to the Guidelines is consistent with other aspects of my job. |
| **Goals** | 1. Compared to my other tasks, offering more outdoor time according to the Guidelines is a higher priority on my agenda. |
|  | 1. I set achievable short-term goals when offering more time outdoors according to the Guidelines. |
